# Bioinformatics prediction and experimental verification identify MAD2L1 and CCNB2 as diagnostic biomarkers of rhabdomyosarcoma

**DOI:** 10.1186/s12935-021-02347-3

**Published:** 2021-11-27

**Authors:** Tian Xia, Lian Meng, Zhijuan Zhao, Yujun Li, Hao Wen, Hao Sun, Tiantian Zhang, Jingxian Wei, Feng Li, Chunxia Liu

**Affiliations:** 1 Department of Pathology and Key Laboratory for Xinjiang Endemic and Ethnic Diseases, The First Affiliated Hospital, Shihezi University School of Medicine, Shihezi, 832002 China; 2 Department of Pathology, The Second Affiliated Hospital of Guangzhou Medical University, Guangzhou, 510260 China; 3 Department of Pathology and Medical Research Center, Beijing Chaoyang Hospital, Capital Medical University, Beijing, 100020 China

**Keywords:** Rhabdomyosarcoma, MAD2L1, CCNB2, Bioinformatics, The tumor microenvironment

## Abstract

**Background:**

Rhabdomyosarcoma (RMS) is a malignant soft-tissue tumour. In recent years, the tumour microenvironment (TME) has been reported to be associated with the development of tumours. However, the relationship between the occurrence and development of RMS and TME is unclear. The purpose of this study is to identify potential tumor microenvironment-related biomarkers in rhabdomyosarcoma and analyze their molecular mechanisms, diagnostic and prognostic significance.

**Methods:**

We first applied bioinformatics method to analyse the tumour samples of 125 patients with rhabdomyosarcoma (RMS) from the Gene Expression Omnibus database (GEO). Differential genes (DEGs) that significantly correlate with TME and the clinical staging of tumors were extracted. Immunohistochemistry (IHC) was applied to validate the expression of mitotic arrest deficient 2 like 1 (MAD2L1) and cyclin B2 (CCNB2) in RMS tissue. Then, we used cell function and molecular biology techniques to study the influence of MAD2L1 and CCNB2 expression levels on the progression of RMS.

**Results:**

Bioinformatics results show that the RMS TME key genes were screened, and a TME-related tumour clinical staging model was constructed. The top 10 hub genes were screened through the establishment of a protein–protein interaction (PPI) network, and then Gene Expression Profiling Interactive Analysis (GEPIA) was conducted to measure the overall survival (OS) of the 10 hub genes in the sarcoma cases in The Cancer Genome Atlas (TCGA). Six DEGs of statistical significance were acquired. The relationship between these six differential genes and the clinical stage of RMS was analysed. Further analysis revealed that the OS of RMS patients with high expression of MAD2L1 and CCNB2 was worse and the expression of MAD2L1 and CCNB2 was related to the clinical stage of RMS patients. Gene set enrichment analysis (GSEA) revealed that the genes in MAD2L1 and CCNB2 groups with high expression were mainly related to the mechanism of tumour metastasis and recurrence. In the low-expression MAD2L1 and CCNB2 groups, the genes were enriched in the metabolic and immune pathways. Immunohistochemical results also confirmed that the expression levels of MAD2L1 (30/33, 87.5%) and CCNB2 (33/33, 100%) were remarkably higher in RMS group than in normal control group (0/11, 0%). Moreover, the expression of CCNB2 was related to tumour size. Downregulation of MAD2L1 and CCNB2 suppressed the growth, invasion, migration, and cell cycling of RMS cells and promoted their apoptosis. The CIBERSORT immune cell fraction analysis indicated that the expression levels of MAD2L1 and CCNB2 affected the immune status in the TME.

**Conclusions:**

The expression levels of MAD2L1 and CCNB2 are potential indicators of TME status changes in RMS, which may help guide the prognosis of patients with RMS and the clinical staging of tumours.

## Background

Rhabdomyosarcoma (RMS) is a malignant soft-tissue tumour that in children, accounting for approximately 6.5% of childhood tumours [1–3]. RMS can be categorised into three main histological types, namely, embryonal RMS (ERMS), alveolar RMS (ARMS), pleomorphic RMS (PRMS) [4, 5]. RMS is highly malignant with unclear pathogenesis; its prognosis is poor and closely related to clinical stage, tumour size, and pathological type [2, 6]. Current clinical treatment approaches for RMS include chemotherapy, radiotherapy and surgery, but their therapeutic effect is limited [3]. Therefore, the pathogenesis of RMS and new treatment strategies need to be explored urgently.

The tumour microenvironment (TME) has been reported to be associated with tumour development, metastasis, and prognosis [7–9]. It is the internal environment of the tumour, and its components include an extracellular matrix as well as endothelial, immune and mesenchymal cells [10]. Immune, mesenchymal, and endothelial cells secrete different cytokines that directly act on tumour cells. Complex and dynamic interactions occur between tumour cells and the TME during tumour development. The cellular regulatory networks and inhibitory molecular networks in the TME affect the occurrence and development of tumours through metabolic reprogramming and tumour infiltration of immune cells [11, 12]. A study detected the expression of immune cells in 50 patients with soft tissue sarcoma. CD3+ (tumour-infiltrating lymphocyte), CD4+ (helper T lymphocyte), CD8+ (cytotoxic T lymphocyte) and FOXP3+ (Treg lymphocytes) can be detected in 98% of biopsy tissues, and macrophages can be detected in 90% of patients. Low levels of CD3+ and CD4+ lymphocytes are associated with a good prognosis [13]. Meanwhile, immunosuppressed individuals present with a higher risk to develop soft tissue malignancies, and tumor infiltration of immune cells affects disease outcome [14, 15]. Some studies have shown the matrix degradation in the TME is the basis for tumour occurrence and aggressiveness, secretion of growth factors, induction of cell migration, and promotion of angiogenesis [16]. The activities of MMP-1, MMP-2, and MMP-9 are upregulated in alveolar RMS (ARMS) compared with embryonal RMS (ERMS), and these enzymes may be one of the factors causing aggressive alveolar subtypes [17]. MMP-1 degrades bone extracellular matrix to promote osteosarcoma metastasis [18]. Thus, the immune cell infiltration and extracellular matrix degradation in the TME may be highly important for the growth, metastasis, and prognosis of RMS. Therefore, the dynamic regulation of stromal and immune components in the TME cannot be ignored, which can serve as a new therapeutic strategy for RMS.

Apart from the TME, microarray analysis has received increasing attention in medical oncology [19]. This technique not only assesses the difference between the genetic and epigenetic changes induced by tumours but also determines biomarkers for disease diagnosis and treatment [19]. ESTIMATE and CIBERSORT calculation methods are important means to measure the ratio of immune infiltrating cells and the ratio of stromal and immune components in various tumors [20–23], although its utility on RMS have not been fully revealed. In the presented article, we proposed a strategy that combined bioinformatics prediction and experiment to evaluate potential indicators of TME status changes in RMS, which could represent a new and attractive anti-cancer strategy.

## Materials and methods

### Collection of data and tissue samples

We downloaded the RMS array sequencing data set from the GEO database (https://www.ncbi.nlm.nih.gov/geo/) and selected the gene expression profile of GSE92689. After deleting samples with incomplete information, 125 tumour samples from patients with RMS were obtained for subsequent analysis. In total, 33 paraffin-embedded RMS samples and 11 control striated muscle tissue specimens were selected from the First Affiliated Hospital of Shihezi University School and the First Affiliated Hospital of Xinjiang Medical University, China. Inclusion criteria for RMS patient samples: (1) clinically and pathologically diagnosed as RMS (2) primary (3) untreated. Exclusion criteria for RMS patients: (1) There are serious systemic diseases such as malignant tumors other than RMS before admission. (2) Participate in any drug trial before admission. (3) Lack of clinicopathological data. All subjects provided written informed consent. This research was performed in compliance with the ethical guidelines of the Helsinki Declaration, and was approved by the hospital ethics committee.

### ESTIMATE analysis

The “ESTIMATE” and “limma” software packages in R (v4.0.3) were employed to measure the stromal and immune scores of RMS patients. The scores were presented in 3 types: stromal, immune, and ESTIMATE scores. The scores were utilized to measure the ratio of stromal or immune components in the TME. The lesser the scores, the lower the ratios of the two components. Finally, each sample was divided into low (< median) or high (> median) scoring group.

### Stromal, immune and ESTIMATE scores with clinical stages

R language was employed to analyse the relationship between clinical staging data and stromal, immune and ESTIMATE scores. Kruskal–Wallis rank sum or Wilcoxon rank sum test was performed to compare the differences between two groups. A p-value of < 0.05 was deemed statistically significant.

### Generation of DEGs between the high and low scoring groups

The “limma Bioconductor” software package in R (v4.0.3) was employed to generate DEGs by comparing high- and low-scoring samples. The “pheatmap” software package in R (v4.0.3) was utilized to construct a DEG heatmap. Venn diagrams were created to compare upregulated and downregulated crossover genes associated with immune/matrix scores. The threshold conditions were as follows: |log2 fold change (log2FC) | > 1.0, and false discovery rate (FDR) < 0.05.

### Functional enrichment analysis

Kyoto Encyclopedia of Genes and Genomes (KEGG) pathway and Gene Ontology (GO) analyses were conducted on 593 DEGs via the “clusterProfiler,” “enrichplot,” and “ggplot2” software packages in R (v4.0.3). Statistical significance was considered at p-value < 0.05.

### Protein–protein interaction (PPI) network analysis

A PPI network was constructed by the STRING database (https://string-db.org/). Then, Cytoscape (v3.7.2) was used for reconstruction. The CytoHubba Cytoscape plug-in was used to identify the core genes on the basis of PPI network, and the confidence of the interactive relationship between nodes in the network was larger than 0.95.

### Relationship between the top 10 hub genes in PPI network and overall survival (OS)

The top 10 hub genes chosen from the PPI network were validated by Gene Expression Profiling Interactive Analysis (GEPIA, http://gepia.cancer-pku.cn/), which showed good performance for estimating 5-year OS. The expression levels of the hub genes were compared between sarcoma patients and control samples, and the OS Kaplan–Meier survival curve was established. Survival analysis was carried out using the survival package in R, and statistical significance was considered at p < 0.05.

### Gene expression level with clinical staging

Data on the clinicopathological features of RMS patients were retrieved from GSE92689. R language was employed to analyse the relationship between clinical staging data and gene expression. Kruskal–Wallis rank sum or Wilcoxon rank sum test was performed to compare the differences between two groups. P-values of < 0.05 were deemed statistical significance.

### Gene set enrichment analysis (GSEA)

A collection of C2 KEGG gene sets (v7.2) was retrieved from the Molecular Signatures database as the target set used by GSEA, and GSEA (v4.0.3) was conducted to elucidate the molecular mechanism of low-expression and high-expression populations. Upregulation and downregulation ways were obtained. Statistical significance was considered at FDR < 0.05.

### Immune cell profile

The immune cell composition in tumour tissue was estimated using CIBERSORT (https://cibersort.stanford.edu/about.php) to assess the difference in the infiltration of 22 immune cells between high- and low-expression groups. Statistical significance was considered at p-value < 0.05.

### Culture and transfection of RMS cells

RMS cell lines (PLA802, RH30 and RD) were supplied by Biotechnology Co., Ltd. (Fu Xiang, Shanghai, China). The normal skeletal muscle cell line (HSKMC) was stored in our laboratory. All cells were cultured in DMEM (Gibco, USA) supplemented with 10% FBS (Gibco, USA) and 1% penicillin–streptomycin (Solarbio, China), and maintained at 37 °C with 5% CO_2_. Lipofectamine 2000 (Life Technologies, USA) was transiently transfected with RMS cells for 24 h in compliance with the manufacturer’s instructions. The siRNA sequences included si-h-MAD2L1, 5ʹ-GGGUCCAAAGUUGAGUGAGUCUUGA-3ʹ and si-h-CCNB2, 5ʹ-CAAGAATGTGGTGAAAGTA-3ʹ.

### Immunohistochemistry (IHC) and IHC assessment

The main antibodies used for IHC were as follows: rabbit anti-CCNB2 (Ab185622, 1:100; Abcam) and rabbit anti-MAD2L1 (Ab97777, 1:1200; Abcam). Paraffin-embedded RMS tissue sections were taken, and IHC staining was performed. The sections were deparaffinized, rehydrated with xylene, and washed with graded alcohol and PBS. After heating for 15 min in citric acid buffer (pH 6.0), antigen retrieval was conducted. TBS/H_2_O_2_ was used to block endogenous peroxidase. After incubation with anti-human CCNB2 and MAD2L1 primary antibodies, the sections were exposed to goat anti-rabbit antibodies at 37 °C for 30 min.

IHC staining was assessed by two independent pathologists with no knowledge of patient characteristics. The staining results of MAD2L1 or CCNB2 were evaluated by staining intensity and degree. The scoring system was as follows: 0 (no staining), 1 (shallow yellow), 2 (brownish yellow), and 3 (dark brown). The proportions of positive staining cells were scored as follows: 0 (0%), 1 (< 25%), 2 (25–75%), and 3 (> 75%). The final scores of < 4 and ≥ 4 was deemed as low and high expression, respectively [24, 25].

### Western blot analysis

The main antibodies used for Western blot were as follows: rabbit anti-CCNB2 (Ab185622, 1:1000; Abcam), rabbit anti-MAD2L1 (Ab97777, 1:1000; Abcam) and mouse anti-β-actin (IE9A3, 1:800; China). The secondary antibody was peroxidase-conjugated goat anti-mouse/rabbit IgG (ZB-2305/2301, 1:10,000; ZSGB). Approximately 48 h after transfection, RMS cell lysis was performed, and the total protein was isolated in RIPA buffer (Solarbio). After electrophoresis, the protein molecules on the gel were electrically transferred to PVDF membranes (Solarbio), immersed in blocking solution (5% non-fat milk/0.1% Tween-20) for 2 h, and then exposed to anti-CCNB2 and anti-MAD2L1 at 4 °C overnight. On the next day, the membrane was exposed the corresponding secondary antibodies at room temperature (RT) for 2 h.

### Immunofluorescence (IF) procedure

The main antibodies used for IF were as follows: rabbit anti-CCNB2 (Ab185622, 1:80; Abcam) and rabbit anti-MAD2L1 (Ab97777, 1:100; Abcam). The secondary antibody was peroxidase-conjugated goat anti-rabbit IgG (ZB-0311, 1:1000; ZSGB). The slides were fixed with 4% paraformaldehyde in the culture plate for 15 min, immersed three times in PBS, and permeated with 0.5% Triton X-100 (prepared in PBS) at RT for 20 min. Serum blocking was performed for 30 min at RT, and the blocking solution was absorbed with an absorbent paper. A sufficient volume of diluted primary antibody was added into each slide. After transferring into a humid box, the slide was incubated overnight at 4 °C. On the next day, the slide was exposed to fluorescent secondary antibodies for 1 h. DAPI was added dropwise to the coverslips, and then incubated for 5 min in the dark. The specimens were stained with nuclei, and then examined using a fluorescence microscope (Olympus BX51, Japan).

### CCK8 assays

Cell Counting Kit-8 (CCK8; Dojindo, Japan) analysis was conducted to measure cell toxicity and proliferation. Tumour cells (1 × 10^4^ cells/well) were grown in a 96-well plate. After transfection or addition of inhibitors at approximately 0, 24, 48 and 72 h, the absorbance of the solution was recorded at 450 nm.

### 5-Ethynyl-2′-deoxyuridine (EdU) staining

The transfected tumour cells (1 × 10^5^ cells/well) were grown in a 12-well plate. The cells were labeled with the EdU kit (KGA337, KeyGen BioTECH, China) and photographed under a fluorescence microscope.

### Acridine orange staining

Acridine Orange (AO) and Evans Blue (EB) at 1 mg each were dissolved in 10 mL of pH 7.2 PBS to prepare a 100 µg/mL stock solution. The transfected tumour cells (1 × 10^6^ cells/well) were grown in a 6-well plate. The same amount was mixed before use and then set aside. A 100 µL aliquot of the pre-observed cell suspension that has been cultured and incubated with samples was added with 4 µL of AO/EB dye and mixed well. A glass slide was placed with a drop of the above mixture and then covered. The staining results were visualized using a fluorescence microscope.

### TUNEL staining

TUNEL apoptosis detection kit was purchased from Shanghai Biyuntian Biotechnology. The transfected tumour cells (1 × 10^5^ cells/well) were grown in a 12-well plate. The transfected cells were fixed in 4% paraformaldehyde (Solarbio) for approximately 30 min, and then washed with PBS three times. Each well was added with TUNEL detection solution (50 µL) and then incubated at 37 °C for 1 h. Images were subsequently collected.

### Flow cytometry of apoptosis

The transfected cells were collected in a six-well plate and washed three times with PBS. Then, Annexin V-FITC (5 µL) and PI staining solution (10 µL) were added to each test tube, and the cells were analysed using a flow cytometer (Partec, Germany).

### Cell cycle analysis

At 24 h after siRNA interference, the cells were collected, washed with PBS, and then fixed with 75% alcohol in a refrigerator at 4 °C overnight. The cells lines were incubated with RNaseA for 30 min before detection. After staining with PI, the cells were analysed by flow cytometry.

### Transwell assay

Matrigel gel was diluted with serum-free cell culture medium at a ratio of 1:8 at 4 °C. Then, 100 µL of this mixture was applied evenly on the surface of the polycarbonate membrane of the upper chamber and placed at 37 °C for 0.5–1 h. Cells in the log phase were harvested and washed with PBS. The density of cells was adjusted to 1 × 10^5^ cells/well. Then, the upper and lower chambers were added with 100 µL of the cell suspension and 600 µL of 10% FBS, respectively. After being placed in the incubator for 24–48 h, the cells were subjected to fixing, staining and counting.

### Statistics

SPSS v20.0 was employed for statistical analysis. Statistical significance was considered at p < 0.05. Relationships of stromal, immune and ESTIMATE scores with tumor staging were analyzed using the Kruskal–Wallis rank sum test. Differentiated expression of genes in the normal and tumor sample were analyzed using the Wilcoxon rank sum test. Multivariate analyses for OS were performed using the Cox proportional hazards model. The correlation of MAD2L1 and CCNB2 expression with clinicopathological staging characteristics were analyzed using the ANOVA test. A Chi-square test was used to analyze the associations between protein expressions and patient characteristics. Independent sample t-test was used to compare the differences between the two groups of different cells. The correlation of immune cell proportion with the MAD2L1 and CCNB2 expression were analyzed using the Pearson’s correlation coefficient. All graphics were drawn using the GraphPad Prism v8.0.

## Results

### Stromal scores are related to clinical staging in RMS patients

**Fig. 1:**
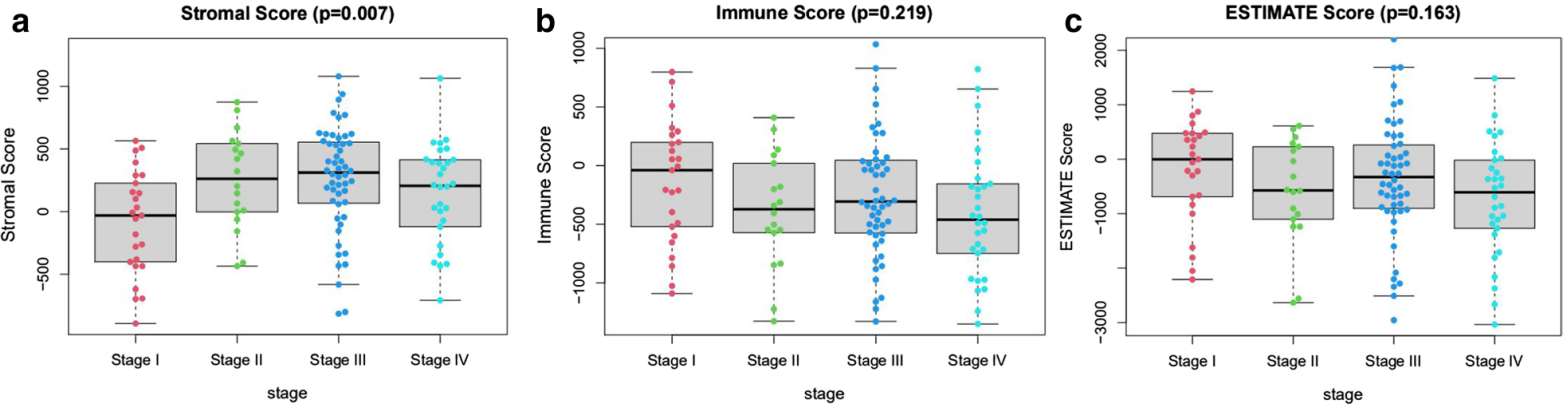
Relationships of stromal and immune scores with the clinicopathological staging features of RMS patients. **a**–**c** Distributions of stromal, immune and ESTIMATE scores in different stages. P-value = 0.219, 0.007, and 0.163, respectively, via Kruskal–Wallis rank sum test

The RNA-Seq and clinical data of 125 RMS patients were retrieved from the GEO database, and the tumour samples were analysed using the ESTIMATE algorithm. The ranges of stromal, immune and ESTIMATE scores were − 893.87–1079.43, − 1350.06–1034.71 and − 3037.15–2202.51, respectively. All 125 samples were classified as high and low scoring groups based on their median scores. A high immune or stromal score indicates immunity in the TME or a large number of stromal components, respectively. The ESTIMATE score was the sum of stromal and immune scores, which represented the combination of these two components in the TME. As shown in Fig. 1a, the RMS cases at Stage III subgroup had the highest average stromal score, followed by Stage II and IV, while the Stage I samples had the lowest stromal score, indicating that stromal scores are meaningful in the correlation of subgroup classification (p = 0.007). However, the immune scores and ESTIMATE were not significantly correlated with clinical staging (Fig. 1b, c). These results indicated that the stromal component in the TME was appropriate for reflecting the clinical stage of RMS patients.

### Overall functional DEGs shared by stromal and immune scores are associated with the enrichment of stromal-related genes

**Fig. 2:**
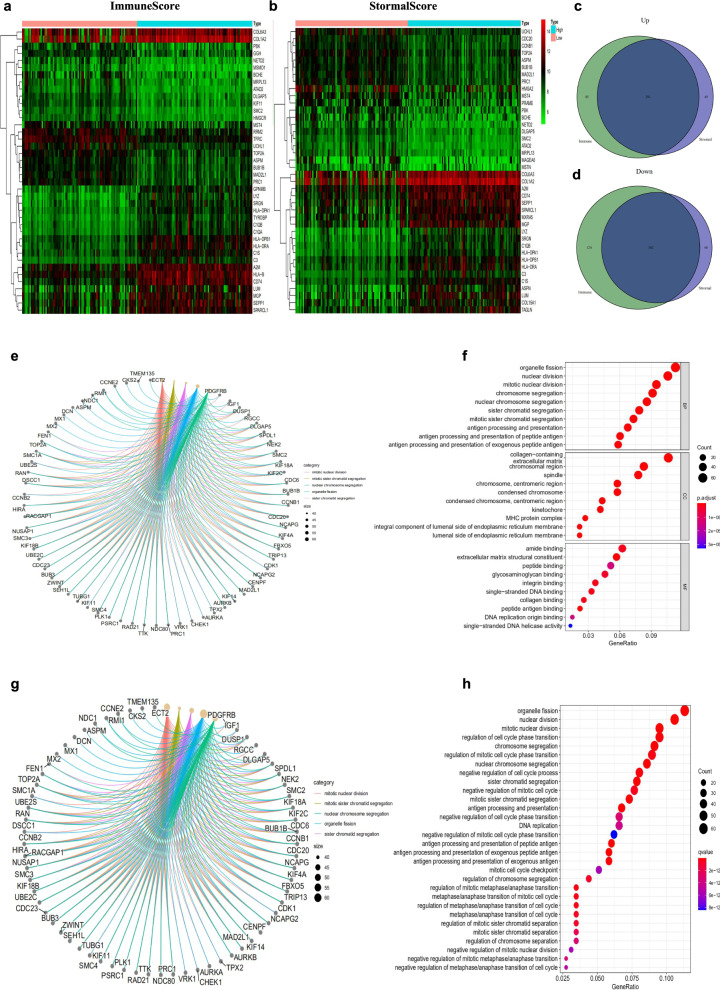
Heatmaps and Venn plots for the DEGs between high and low stromal/immune scoring groups, and functional enrichment of DEGs. **a** Heatmap for DEGs between high and low immune scoring groups. **b** Heatmap for DEGs between low and high stromal scoring groups. **c**, **d** Venn diagram shows the overlapped DEGs between stromal and immune scoring groups. **e** Circos plot reveals the association between DEGs and top GO enrichment terms. **f** GO analysis shows the BP, CC and MF terms for 593 DEGs. **g** KEGG analysis reveals the 30 pathways for 593 DEGs. **h** Circos plot indicates the association between DEGs and top KEGG pathways. *DEGs* differentially expressed genes, *GO* gene ontology, *MF* molecular function, *CC* cellular component, *BP* biological process, *KEGG* Kyoto Encyclopedia of Genes and Genomes

The levels of gene expression in low and high scoring groups were compared using |log2FC| > 1.0 and FDR < 0.05 as the screening criteria to ascertain the accurate changes of the gene profiles of stromal and immune components in the TME. In total, 802 DEGs were obtained from the immune score, of which 376 genes were upregulated, while the remaining 426 genes were downregulated (Fig. 2a). Meanwhile, 682 DEGs were associated with the stromal score, including 340 upregulated genes and 342 downregulated genes (Fig. 2b). Because the crosstalk between stromal cells and immune cells in the TME affects the occurrence and development of tumors [26–28]. Therefore, we analyzed both immune and stromal DEGs finding the shared DEGs. The Venn diagram takes the intersection of genes related to the stromal and immune scores. The analysis showed that 291 upregulated genes and 302 downregulated genes were overlapped in the stromal and immune scores (Fig. 2c, d). Thus, these DEGs (593 genes in total) can play an essential role in regulating the TME. Afterwards, functional enrichment analysis was performed on these 593 important DEGs. GO enrichment analysis showed that the top three enriched biological process (BP) terms were “mitotic nuclear division”, “nuclear division” and “organelle fission”. The top three enriched cellular component (CC) terms were “collagen-containing,” “extracellular matrix,” and “chromosomal region.” The first three enriched molecular function (MF) terms were “amide binding”, “extracellular matrix structural constituent” and “peptide binding” (Fig. 2e, f). The KEGG results also showed that “organelle fission”, “regulation of mitotic cell cycle phase transition”, “regulation of cell cycle phase transition”, “nuclear division” and other signal pathways were related (Fig. 2g, h). Therefore, the overall function of DEG was associated with the functions of cell differentiation and cycle regulation, implying that the involvement of stromal cells was the prominent feature of the TME in RMS.

### Network analysis of the overlapped DEG and validation with TCGA database

**Fig. 3:**
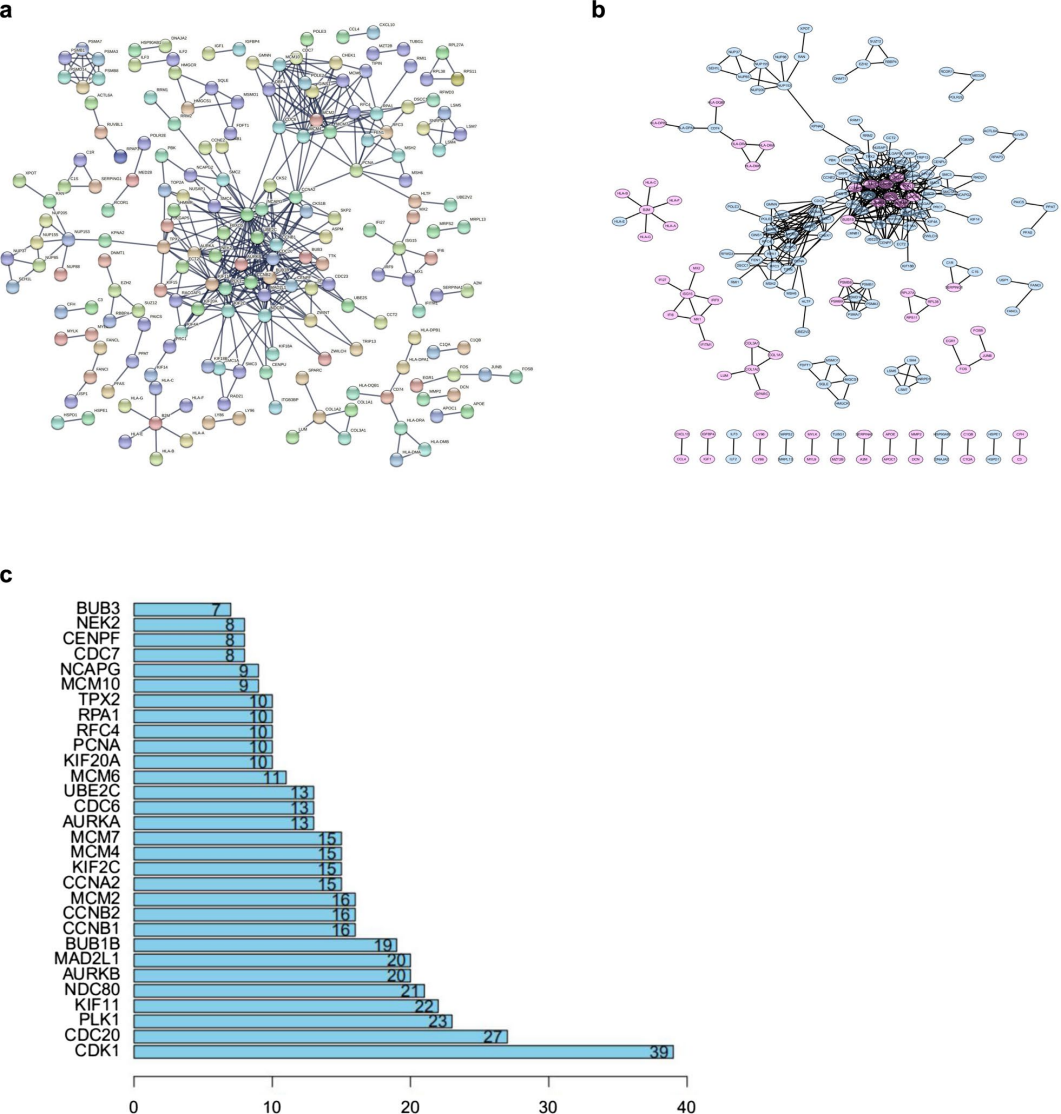
Establishment of PPI networks. **a** Interaction networks of differentially expressed genes containing various nodes with confidence score > 0.99. **b** PPI network visualization with Cytoscape software. Purple means upregulation, and blue means downregulation. **c** The 30 most significant genes identified by the amount of nodes

A PPI network of 593 shared DEGs in the stromal and immune score groups was constructed using STRING network analysis tools to further explore its potential mechanism (Fig. 3a). Visualization using Cytoscape software showed that the PPI network is composed of 189 nodes and 836 edges (Fig. 3b). We identified 30 most significant genes in the PPI network, including CDK1, CDC20, PLK1, KIF11, NDC80, AURKB, MAD2L1, BUB1B, CCNB1 and CCNB2 as the top 10 hub genes (Fig. 3c).

**Fig. 4:**
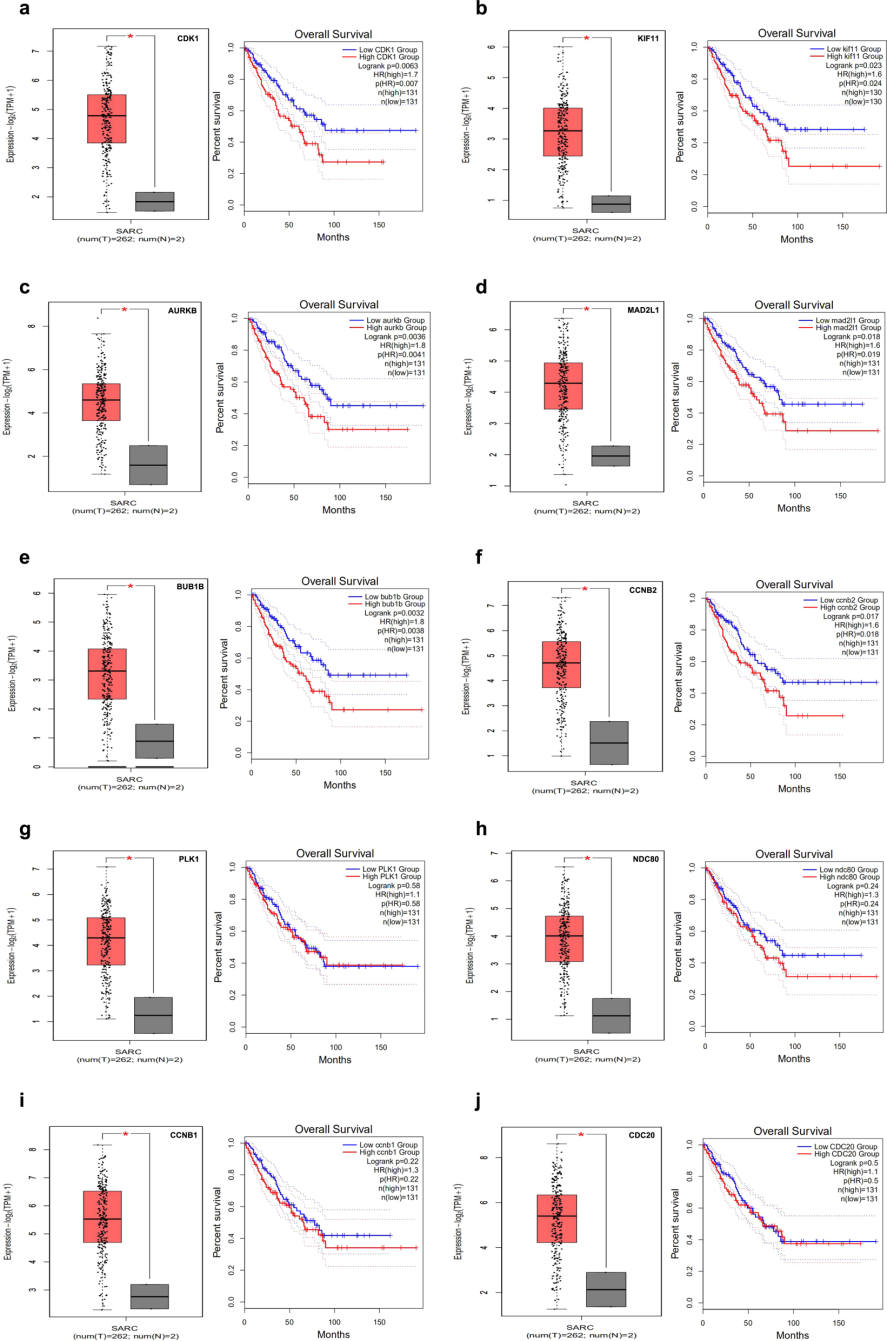
The top 10 hub genes in PPI network are differentially expressed between sarcoma and control tissues, and comparison of OS between patients with high and low expression. **a** CDK1, **b** KIF11, **c** AURKB, **d** MAD2L1, **e** BUB1B, **f** CCNB2, **g** PLK1, **h** NDC80, **i** CCNB1, **j** CDC20. *P < 0.05. *HR* hazard ratio, *OS* overall survival, *SARC* sarcoma

Some studies have shown that the TCGA data set can be utilized to verify the associations between the expression of genes and OS in a variety of sarcoma [29–33]. Therefore, we used a similar method to verify the associations between OS and the expression of the top 10 hub genes in the PPI network. First, we found that the levels of CDK1, CDC20, PLK1, KIF11, NDC80, AURKB, MAD2L1, BUB1B, CCNB1, and CCNB2 in sarcoma tissues were upregulated compared to those in control tissues by GEPIA (http://gepia.cancer-pku.cn/). Whether or not the expression levels of these 10 hub genes are related to the OS of sarcoma patients was further studied. Our analysis of sarcoma data in TCGA by GEPIA showed that high levels of CDK1, KIF11, AURKB, MAD2L1, BUB1B and CCNB2 (Fig. 4a–f) were strongly related to worse OS (P = 0.0063, 0.0023, 0.0036, 0.018, 0.0032 and 0.017, respectively), whereas the four other hub genes (PLK1, NDC80, CCNB1, CDC20) demonstrated no significant association (Fig. 4g–j). Thus, we determined these six hub genes as the object of follow-up research.

### MAD2L1 and CCNB2 may be potential indicators of TME status and clinical stage

**Fig. 5:**
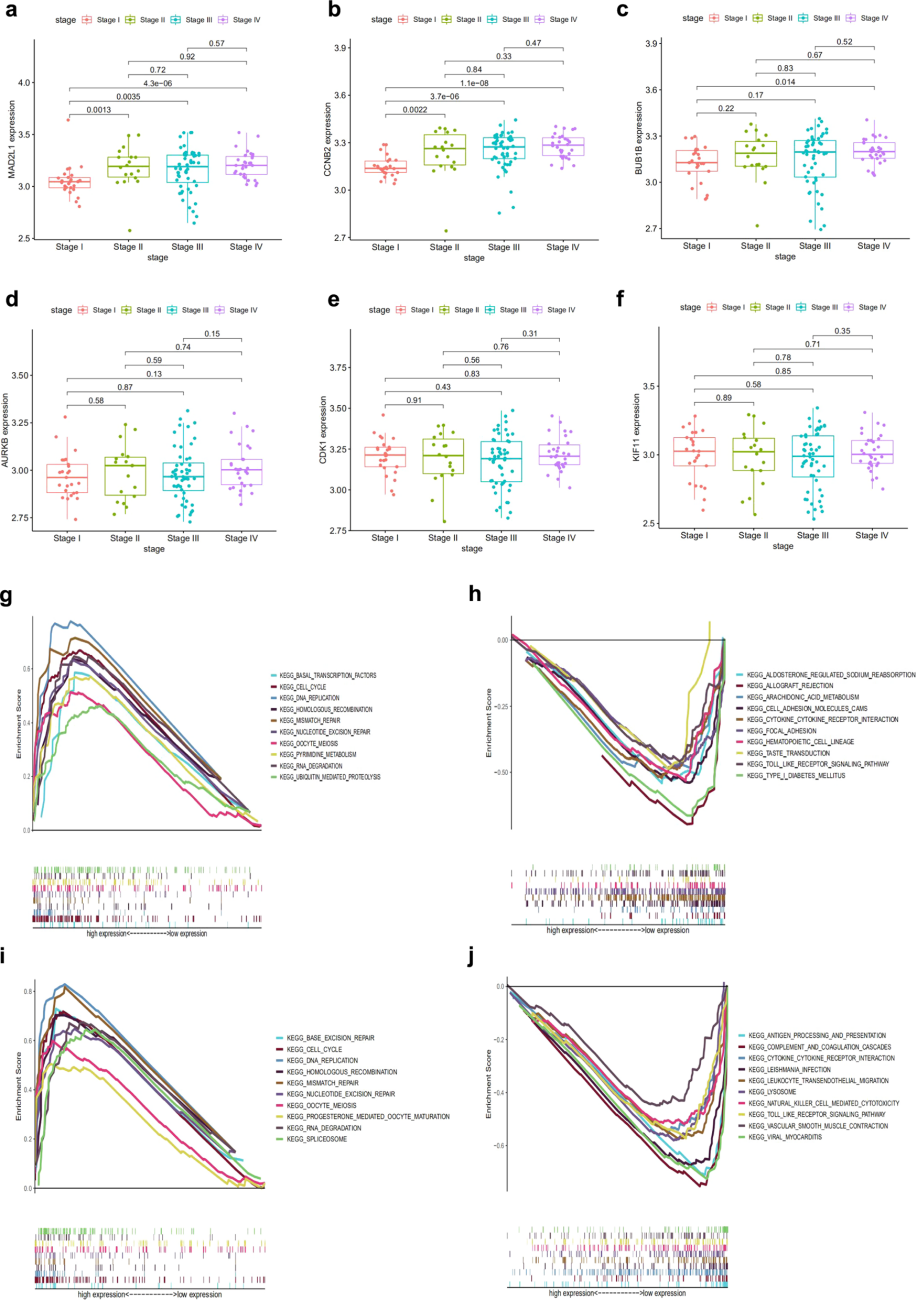
Correlation of six hub genes related to survival overall survival with clinicopathological staging characteristics, and GSEA for samples with low and high expression of MAD2L1 or CCNB2. **a** MAD2L1, **b** CCNB2, **c** BUB1B, **d** AURKB, **e** CDK1, and **f** KIF11. The p value by Kruskal–Wallis rank sum test. **g** KEGG gene-set enrichment in high MAD2L1 expression group. Each line denotes a gene set with unique colour. Significance level = p-value < 0.05 and FDR q < 0.05. **h** KEGG gene-set enrichment by the low MAD2L1 expression sample. **i** KEGG gene-set enrichment by the high CCNB2 expression sample. **j** KEGG gene-set enrichment by the low CCNB2 expression sample

As mentioned above, CDK1, KIF11, AURKB, MAD2L1, BUB1B and CCNB2 were highly expressed in sarcoma and were markedly related to worse OS. Then, we further studied their relationship with the clinical stage of RMS. The findings demonstrated that the expression of MAD2L1 and CCNB2 were associated with the clinical stage of RMS patients. (Fig. 5a, b), whereas the four other hub genes were not related the clinical stage of RMS patients (Fig. 5c–f).

Considering the expression MAD2L1 and CCNB2 was correlated with clinical stage in RMS patients, we further determined the differences in enrichment pathways between low- and high-expression groups, and performed GSEA testing. Our results demonstrated that the genes in MAD2L1 high-expression group were involved in DNA replication, mismatch repair, and cell cycle (Fig. 5g). By contrast, the MAD2L1 low-expression group was related to allograft rejection, type 1 diabetes mellitus, and cell adhesion molecules (Fig. 5h). The genes in CCNB2 high-expression group were mainly associated with DNA replication, mismatch repair and cell cycle (Fig. 5i). By contrast, the genes in CCNB2 low-expression group were associated with complement and coagulation cascades, viral myocarditis, and antigen processing and presentation (Fig. 5j). These pathways were mostly related to the mechanism of RMS metastasis and recurrence. Therefore, MAD2L1 and CCNB2 may be potential indicators of TME status and clinical stage.

### MAD2L1 and CCNB2 were highly expressed in RMS tissue and cell lines

**Fig. 6:**
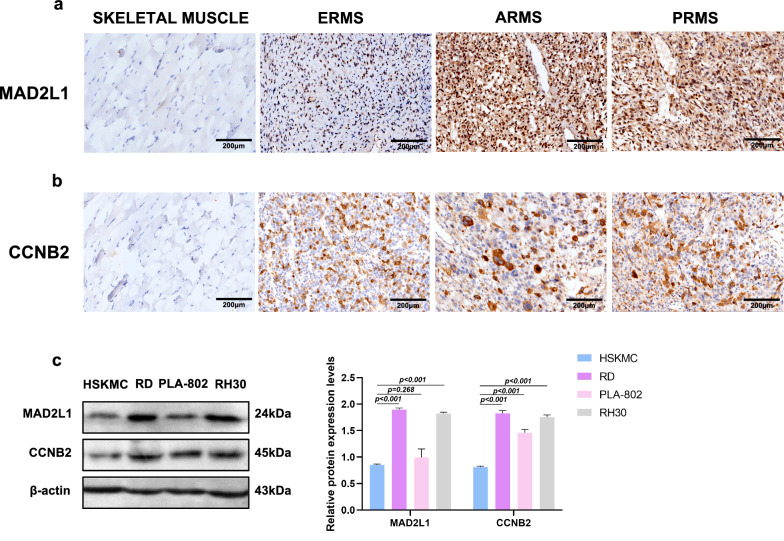
Expression of MAD2L1 and CCNB2 in RMS tissues and cells. **a** IHC analysis of MAD2L1 demonstrates strong nuclear expression in patients with RMS. Negative control, ERMS, ARMS, PRMS (all ×200). **b** IHC analysis of CCNB2 demonstrates cytoplasm or nuclear expression in patients with RMS. Negative control, ERMS, ARMS, PRMS (all ×200). **c** Western blot shows the expression of MAD2L1 and CCNB2 in normal skeletal muscle cells (HSKMC) and RMS cell lines (RD, PLA-802, RH30)

Next, we performed IHC to test the expression of MAD2L1 and CCNB2 in RMS tissue. The staining site of MAD2L1 was mainly in the nucleus, and no MAD2L1 expression (0%, 0/11) was detected in 11 control skeletal muscle tissues. The expression rate of MAD2L1 in 33 cases of RMS was 90.9% (30/33) (Fig. 6a are skeletal muscle tissues, ERMS, ARMS, and PRMS tissues, respectively). The expression rates of MAD2L1 were markedly higher in RMS group that in normal control group (p < 0.001). No obvious difference was found between the expression of MAD2L1 and clinical data (Table 1). Positive cytoplasmic staining was observed for CCNB2, and it was not expressed in 11 control skeletal muscle tissues. The expression rate of CCNB2 in 33 cases of RMS was 100% (33/33) (Fig. 6b are skeletal muscle tissues, ERMS, ARMS, and PRMS tissues, respectively). The expression rates of CCNB2 were noticeably higher in RMS group than in normal control group (p < 0.001). According to the results of Chi-square test, there was an obvious difference in CCNB2 expression between patients with different tumour sizes (p < 0.001, Table 1). RMS patients with tumour diameters > 5 cm were more susceptible to high CCNB2. Western blot results showed that compared with normal skeletal muscle cells (HSKMC), MAD2L1 was upregulated in RMS cell lines (RD and RH30). CCNB2 was also upregulated in the three cell lines (RD, PLA-802 and RH30) (Fig. 6c). These results are similar to those derived from GEO and TCGA databases, indicating the high reliability and validity of our findings.

**Table 1:** Association between MAD2L1 and CCNB2 protein expression and patient clinical characteristics

Patient characteristics	n (%)	MAD2L1		P-value	CCNB2		P-value
		Low expression, n (%)	High expression, n (%)		Low expression, n (%)	High expression, n (%)	
Gender							
Male	21 (63.6)	9 (42.9)	12 (57.1)	0.457	8 (38.1)	13 (61.9)	0.259
Female	12 (36.4)	3 (25)	9 (75)		2 (16.7)	10 (83.3)	
Age (years)							
≤ 5	7 (21.2)	5 (71.4)	2 (28.6)	0.071	4 (57.1)	3 (42.9)	0.161
> 5	26 (78.8)	7 (26.9)	19 (73.1)		6 (23.1)	20 (76.9)	
Tumor diameter							
≤ 5 cm	11 (33.3)	4 (36.4)	7 (63.6)	1.000	8 (72.7)	3 (27.3)	0.000^a^
> 5 cm	22 (66.7)	8 (36.4)	14 (63.6)		2 (9.1)	20 (90.9)	
Histology							
ERMS	14 (42.4)	7 (50)	7 (50)	0.427	5 (35.7)	9 (64.3)	0.164
ARMS	12 (36.4)	3 (25)	9 (75)		5 (41.7)	7 (58.3)	
PRMS	7 (21.2)	2 (28.6)	5 (71.4)		0 (0.0)	7 (100.0)	
Location							
Head and neck	9 (27.3)	3 (33.3)	6 (66.7)	1.000	4 (44.4)	5 (55.6)	0.943
Torso and limbs	16 (48.5)	6 (37.5)	10 (62.5)		5 (31.3)	11 (68.7)	
Urinary	5 (15.2)	2 (40)	3 (60)		2 (40)	3 (60)	
Abdominal or retroperitoneal	3 (9)	1 (33.3)	2 (66.7)		1 (33.3)	2 (66.7)	

P < 0.05 indicates a significant association among the variables

^a^Significant difference

### Downregulation of MAD2L1 and CCNB2 inhibits RMS cell growth

**Fig. 7:**
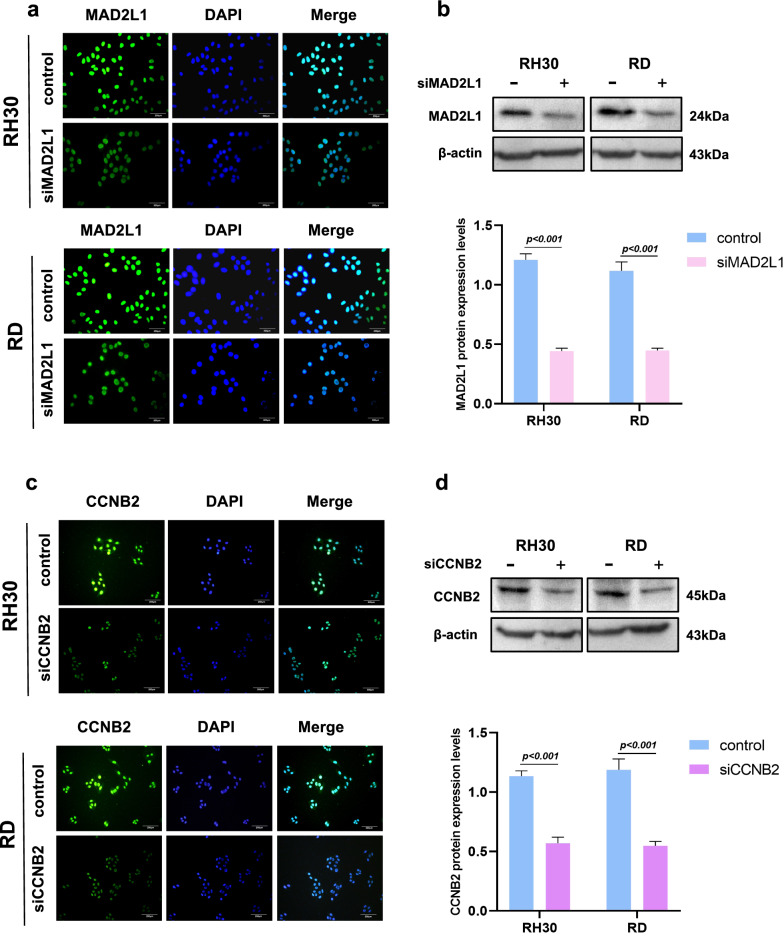
Downregulation of MAD2L1 and CCNB2. **a** IF assessment of RH30 and RD cells transfected with si-MAD2L1 and si-CCNB2. Cells were stained with 4ʹ,6-diamidino-2-phenylindole (blue) and the antibody against MAD2L1 (green), exposed to the corresponding secondary antibody, and analysed with double IF assay. **b** Western blot shows the expression of siMAD2L1. **c** and **d** IF and Western blot shows the expression of siCCNB2

SiRNAs were used to inactivate MAD2L1 and CCNB2 in RMS cells to determine whether or not MAD2L1 and CCNB2 can be used as therapeutic targets for RMS. Immunofluorescence showed that the fluorescence expression intensities of the siMAD2L1 groups were remarkably attenuated compared to those of the control group and western blot findings demonstrated that the protein expression levels of the siMAD2L1 groups were lower than that of the control group (Fig. 7a, b). The results of siCCNB2 groups were similar (Fig. 7c, d). This finding shows that the downregulation of MAD2L1 and CCNB2 is effective and provides a basis for subsequent experiments.

**Fig. 8:**
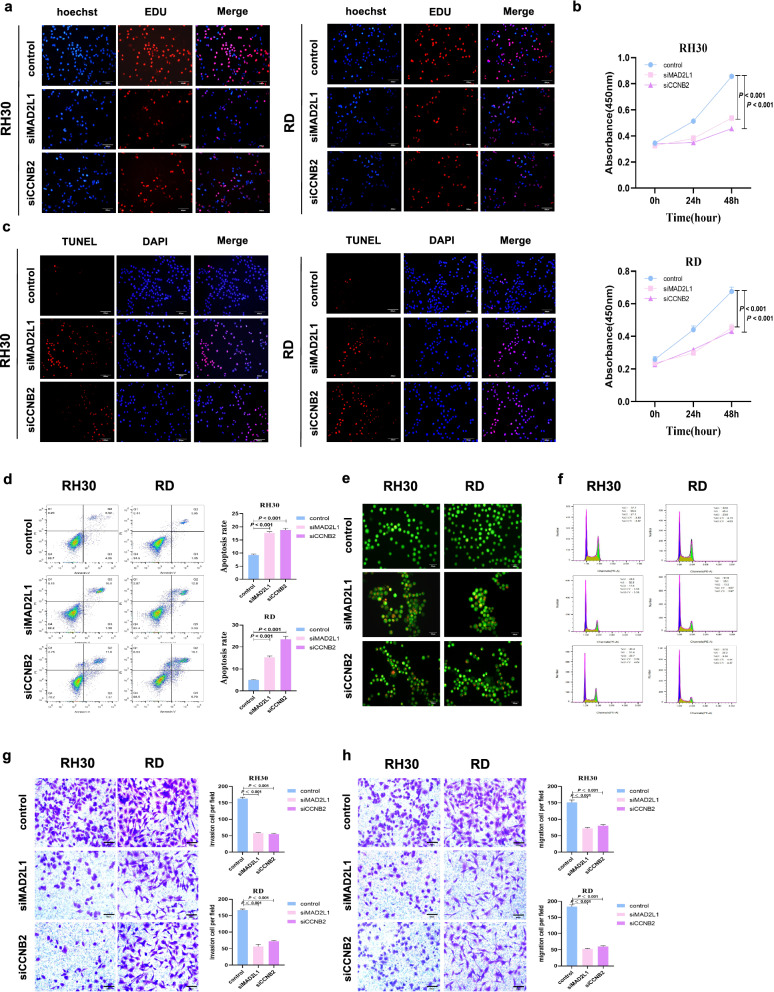
Downregulation of MAD2L1 and CCNB2 inhibits RMS cell growth, invasion and migration as well as promotes cell cycle arrests and cell death. **a** EdU assays showed that RMS cell proliferation was detected. Hoechst staining, EdU labeled cell proliferation (red), total cells (blue), siMAD2L1 and siCCNB2 inhibited cell growth (pink). **b** CCK8 shows the cell proliferation ability. **c** Apoptosis of RH30 and RD cells transfected with small interfering RNA fragments of MAD2L1 and CCNB2 was detected by TUNEL. DAPI staining, all cells (blue), TUNEL-labeled apoptotic cells (red), siMAD2L1 and siCCNB2 promoted cell apoptosis (pink). **d** Rate of apoptosis was analysed by flow cytometry. **e** AO staining apoptotic cells, followed by flow cytometric analysis. **f** Changes of cell cycle. **g**, **h** The migration and invasion capabilities of RMS cells. P-value < 0.05, via independent sample t-test

The results of EdU (Fig. 8a) and CCK8 (Fig. 8b) assays observed that reduced MAD2L1 and CCNB2 expression resulted in the inhibition of the proliferation ability and vitality of RMS cells. TUNEL and flow cytometry assays demonstrated that the downregulation of MAD2L1 and CCNB2 promoted the apoptosis of RMS cells (Fig. 8c, d). AO staining showed that red fluorescent apoptotic cell nucleus increased after the downregulation of MAD2L1 and CCNB2 (Fig. 8e). In addition, GSEA showed that CCNB2 and MAD2L1 are closely related to the cell cycle. Thus, we measured the cell cycle by performing flow cytometry assays. Compared to control group, the proportion of G1-phase cells in siMAD2L1 and siCCNB2 groups increased, while the proportion of G2-phase cells decreased (Fig. 8f), indicating that the decreased MAD2L1 and CCNB2 expression suppressed the growth of RMS cells. Transwell assays showed that the number of tumour cell migration and invasion was markedly lower in siMAD2L1 and siCCNB2 groups than in control group (Fig. 8g, h).

### Correlation of MAD2L1 and CCNB2 with immune cell infiltration

**Fig. 9:**
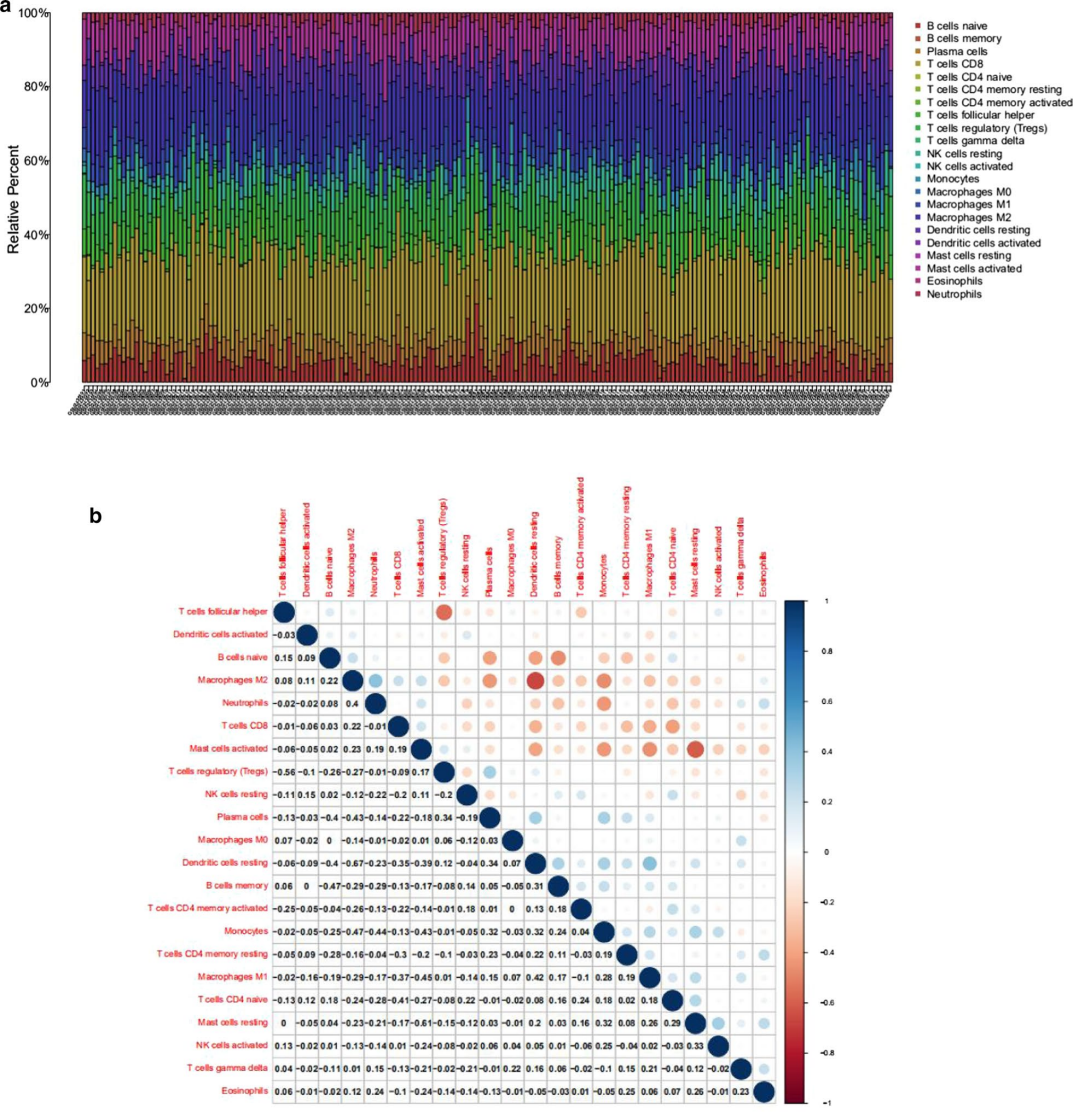
Correlations between the tumour sample and immune cell relative percent. **a** Bar plot indicates the proportion of immune cells (22 types) in RMS samples. **b** Heatmap shows the relationships among the 22 types of immune cells, and the size of each circle indicates the significance level of each association. Blue denotes low and red denotes high. P-value < 0.05, via Pearson’s correlation coefficient

**Fig. 10:**
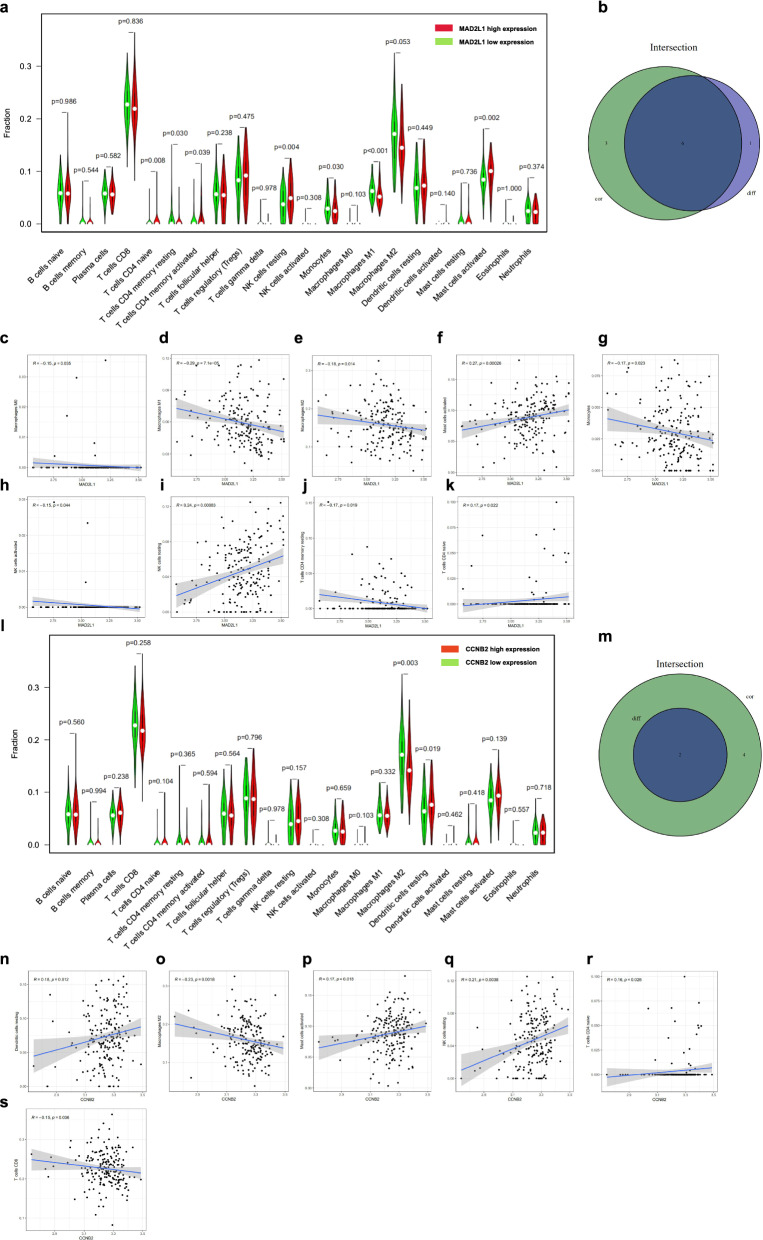
Association between immune cell proportion and MAD2L1 or CCNB2 expression. **a** Violin plot shows the proportion of immune cells (22 types) between control tissues with high and low MAD2L1. P-value < 0.05, via Wilcoxon rank sum test. **b** Venn plot shows the correlation between six types of immune cells and MAD2L1 expression, as codetermined by difference and correlation analysis shown in scatter and violin plots. **c**–**k** Scatter plot shows the association of nine types of immune cell proportion with MAD2L1 expression. Blue line indicates fitted linear model. P-value < 0.05, via Pearson’s correlation coefficient. **l** Violin plot of CCNB2. **m** Venn plot shows two types of immune cells correlated with CCNB2 expression. (N–S) Scatter plot indicates the relationship between six types of immune cells and CCNB2 expression. P-value < 0.05

As mentioned above, our study revealed that the high levels of MAD2L1 and CCNB2 promoted the proliferation, invasion, migration, and inhibition of apoptosis of RMS cells, which may be a potential target for RMS treatment. Whether or not a connection exists among MAD2L1, CCNB2 and immune infiltrating cells was further explored. The CIBERSORT algorithm was employed to analyse the differences and correlation in immune infiltration of 22 types of immune cells between low- and high-expression populations in RMS samples (Fig. 9a, b). Differential expression and correlation analyses were performed for verification, and then the intersection was determined to obtain the common immune cells. Six tumour-infiltrating immune cells (TICs) were found to be associated with MAD2L1 expression. Specifically, three TICs were positively correlated to MAD2L1 expression, including resting NK cells, naïve T cells CD4 and mast cells; while the remaining three TICs were negatively correlated to MAD2L1 expression, including resting memory T cells CD4, monocytes, and macrophages M1 (Fig. 10a–k). Analysis of CCNB2 using the same method revealed that two TICs were related to the expression of CCNB2. Resting dendritic cells were positively correlated to CCNB2 expression. Macrophages M2 negatively correlated with CCNB2 expression, but all the correlations reported were very weak (Fig. 10l–s). Altogether, these findings further confirmed that MAD2L1 and CCNB2 were related to the TME.

## Discussion

RMS is a common malignant tumour in children, and the pathogenesis remains unclear. The prognosis of RMS patients is poor, the treatment effect is limited, a clear targeted therapy drug remains unavailable, and the 5-year OS rate is less than 20% [34–36]. Thus, identifying the diagnostic and prognostic biomarkers of the disease is crucial for reducing mortality and morbidity.

The TME plays crucial roles in tumour initiation and prognosis. Therefore, exploring the key genes that affect TME is beneficial to inhibit tumour metastasis and prognosis, which may be a potential target for tumour treatment. Some studies have shown that matrix degradation in the TME is a key factor in promoting tumour development and invasion. The expression levels of α-dystroglican (an essential complex for the assembly and binding of laminin and basement membrane) are downregulated in RMS and other solid tumours. Although the meaning of these differential levels in tumour biology has not been clarified, the decreased adhesion of these cells to laminin could also lead to increased migration capabilities [37]. The macrophage migration inhibitory factor (MIF) expressed by the RMS cell line interacts with CXCR4 and CXCR7 (RMS cell surface receptors) in the paracrine loop, which can reduce the number of cancer-associated fibroblast infiltration, increase cell adhesion, and promote blood vessel formation. Downregulation of MIF in the RMS cell line leads to large xenografts, high stromal cell support, and high tumour cell count [38]. Although many studies have suggested that changes in the stromal and immune components of TME promote cancer progression, the relationship between key differential genes in the TME and cancer prognosis and tumour stage remain unclear. Therefore, in our study, TME-related genes were screened from the GEO datasets that help predict the OS rate and tumour stage of RMS patients, analysed the biological functions and signalling pathways of relevant DEGs, and evaluated the roles of key genes in regulating immune cell infiltration.

First, the ESTIMATE algorithm was employed to measure the stromal, immune, and estimate scores and then studied the association between these scores and clinical staging in 125 patients with RMS. We observed a correlation between stromal score and clinical staging. Then, we compared the expression levels of genes in the low and high scoring groups, and 593 DEGs were screened. KEGG and GO enrichment analyses showed that these DEGs are associated with the differentiation and cycle regulation of stromal cells, which is consistent with a previous study [39]. However, although the pathogenesis of RMS is unknown, histological studies have shown that the failure of skeletal muscle lineage precursors to exit the cell cycle and fusion of componentized syncytial muscles is an important factor in the development of RMS [40, 41].

Then, we constructed the PPI network and screened the most significant hub genes (CDK1, CDC20, PLK1, KIF11, NDC80, AURKB, MAD2L1, and BUB1B). The overall survival analysis of these genes was performed, and six statistically significant differential genes (CDK1, KIF11, AURKB, MAD2L1, BUB1B, and CCNB2) were obtained. CDK1 promotes the development of lung cancer [42]. It is a promising drug target or prognostic marker for cancer patients, such as hepatocellular carcinoma, lung cancer, and pancreatic ductal adenocarcinoma [43–45]. High expression of KIF11 is associated with the worse prognosis of clear cell renal cell carcinoma [46]. Inhibition of AURKB can suppress the proliferation of osteosarcoma cells [47, 48]. The abnormal expression of MAD2 (also known as MAD2L1) may be related to the pleomorphic morphology and impaired mitoses of soft-tissue sarcoma and the high-grade tumour progression of the TA subgroup [49]. BUB1B accelerates the progression of prostate cancer via the transcriptional modulation of MELK, which can be used as a clinical prognostic factor and drug target for prostate cancer [50]. CCNB2 may responsible for the initiation and progression of liver cancer through the CCNB2/PLK1 pathway and increase the growth and migration of liver cancer cells [51]. miR-335-5p can target the downregulation of CCNB2, thereby inhibiting the occurrence and development of lung adenocarcinoma [52]. In summary, the six genes predicted by our research promote the occurrence of various tumours, and may serve as the diagnostic markers of RMS.

To understand further the role of these six differential genes in RMS, we analysed their correlation with the clinical stage of RMS and finally screened out MAD2L1 and CCNB2. These two genes are correlated with the clinical stage of tumours. Some studies reported that MAD2L1 and CCNB2 are highly expressed in gastric cancer, liver cancer, lung cancer, and other types of cancers; thus, they possibly serve as tumour-promoting genes [51–55], which are consistent with our immunohistochemical results. Our results further revealed that CCNB2 expression is associated with tumour size. Previous studies reported that MAD2L1 and CCNB2 are responsible for cell cycle regulation [56, 57]. Our GSEA analysis revealed the key difference between the high- and low-expression groups; and particularly, the high-expression group is prominently related to the cell cycle pathway. Our cycle analysis results also showed that inhibiting MAD2L1 and CCNB2 blocks cells in the G0/G1 phase, thereby inhibiting RMS cell proliferation. miR-139-5p attenuates the growth, invasion and migration of lung adenocarcinoma cells by targeting MAD2L1 [55]. MAD2L1 can regulate the growth and apoptosis of colorectal cancer cells [58]. Dietary sugar increases the growth of pancreatic cancer cells by increasing MAD2L1 expression [59]. CCNB2, which is upregulated in colorectal cancer, may promote tumour cell growth by accelerating the cell cycle [60]. HMGA induces the overexpression of CCNB2 to promote the development of human pituitary tumours [61]. In this research, inhibiting the expression of MAD2L1 and CCNB2 inhibited the proliferation, invasion, migration, and promotion of apoptosis of RMS cells. Various signs indicate that the upregulation of MAD2L1 and CCNB2 promotes the occurrence and development of RMS.

To study these two genes in more depth, the CIBERSORT calculation method was utilized to measure the proportion of immune infiltrating cells in the RMS sample and analysed the relationship between the proportion of immune infiltrating cells and MAD2L1 and CCNB2. Our findings showed that the expression of MAD2L1 was positively correlated with resting NK cells, naive T cells CD4 and mast cells, while negatively correlated with resting memory T cells CD4, monocytes, and macrophages M1. The expression of CCNB2 was positively correlated with resting dendritic cells and negatively correlated with macrophages M2. Some studies also indicated that MAD2L1 and CCNB2 may be related to immune infiltration [62–65]. These results further support that the levels of MAD2L1 and CCNB2 are closely related to TME. The development of tumours is influenced by various aspects. The role of TME in tumours has become a research hotspot in recent decades. Research on this type of direction provides ideas for the pathogenesis and treatment of RMS.

Although our research revealed some of the functions of genes in the RMS microenvironment, it still has limitations. Since the relationship between TME, MAD2L1, and CCNB2 is complex, Additional studies will be needed to explore the exact mechanisms between TME, MAD2L1, CCNB2, and carcinogenesis of RMS.

## Conclusions

Through bioinformatics analysis, we finally identified two genes related to TME and immune cell infiltration, MAD2L1 and CCNB2. And our results showed that the expression levels of MAD2L1 and CCNB2 correlated with the overall survival of patients with RMS and the clinical stage of the tumor. Finally, our experimental results showed that MAD2L1 and CCNB2 were highly expressed in RMS cells and tissues, downregulation of MAD2L1 and CCNB2 inhibited growth of rhabdomyosarcoma cells. Thus, MAD2L1 and CCNB2 are potential indicators of TME status changes in RMS.

## Data Availability

All data generated or analyzed during this study are included in this published article.
